# Purinergic Signaling on Leukocytes Infiltrating the LPS-Injured Lung

**DOI:** 10.1371/journal.pone.0095382

**Published:** 2014-04-18

**Authors:** Daniela Friebe, Tao Yang, Timo Schmidt, Nadine Borg, Bodo Steckel, Zhaoping Ding, Jürgen Schrader

**Affiliations:** 1 Department of Molecular Cardiology, Heinrich-Heine-University Düsseldorf, Düsseldorf, Germany; 2 Department of Anesthesiology and Intensive Care Medicine, Changhai Hospital, Second Military Medical University, Shanghai, PR China; University of Colorado Denver, United States of America

## Abstract

Extracellular nucleotides and nucleosides have been implicated as important signaling molecules in the pathogenesis of acute lung injury (ALI). While adenosine is known to inhibit T cell activation, little information is available as to ATP and NAD degrading enzymes, the expression of ATP and adenosine receptors/transporters in different T cell subsets. ALI was induced by challenging mice with intra-tracheal instillation of 60 µl (3 µg/g) LPS. After 3 d and 7 d blood, lung tissue and bronchoalveolar lavage was collected and immune cells were analyzed using flow cytometry. The transcriptional phenotype of T helper cells, cytotoxic and regulatory T cells sorted by FACS was assessed by measuring the expression profile of 28 genes related to purinergic signaling using TaqMan Array Micro Fluidic Cards. Catabolism of ATP, NAD and cAMP by activated CD4^+^ T cells was evaluated by HPLC. CD73 was found to be highly abundant on lymphoid cells with little abundance on myeloid cells, while the opposite was true for CD39. After ALI, the abundance of CD39 and CD73 significantly increased on all T cell subsets derived from lung tissue and bronchoalveolar space. Expression analysis in T cell subsets of the lung revealed ATP (*Cd39*, *Cd73*) and NAD (*Cd38*, *Cd157*, *Cd296*, *Pc-1*) degrading enzymes. However, only transcription of *Cd38*, *Cd39*, *Cd73*, *Ent1* and *A2a receptor* was significantly upregulated after ALI in T helper cells. CD4^+^ T cells from injured lung rapidly metabolized extracellular ATP to AMP and adenosine but not NAD or cAMP. These findings show that lung T cells – the dominant cell fraction in the later phase of ALI – exhibit a unique expression pattern of purinergic signaling molecules. Adenosine is formed by T cells at an enhanced rate from ATP but not from NAD and together with upregulated A2a receptor is likely to modulate the healing process after acute lung injury.

## Introduction

The acute respiratory distress syndrome (ARDS), as a result of severe acute lung injury (ALI), is a life-threatening syndrome and the leading cause of morbidity and mortality in critically ill patients [1]. ALI can be caused not only by direct disorders such as pneumonia, aspiration of gastric content, but also indirectly, such as after severe trauma or during sepsis [2]. Besides the influx of a protein-rich edema fluid into the interstitial lung tissue and the bronchoalveolar space due to an increased pulmonary vascular permeability, the excessive infiltration of immune cells is a key feature of ALI [3]–[5]. Intense investigations on the early phase of ALI (1–3 d) revealed inflammatory processes and molecules that initiate the injury [3], [6]–[8]. Factors and mechanisms that contribute to the resolution of the inflammation in the later phase of ALI (5–10 d) remain to be fully elucidated. Currently, no specific therapies are available for ALI and in the clinical setting ALI is often diagnosed after being fully developed. This underlines the need for new therapeutical strategies focusing on the resolution of pulmonary inflammation.

Extracellular nucleotides and nucleosides have been shown to act as important immune modulators [9]. Particularly, adenosine has been demonstrated to be a potent anti-inflammatory mediator in the regulation of several inflammatory conditions [10]. Extracellular adenosine is thought to be formed predominantly by the sequential dephosphorylation of ATP and AMP involving the ecto-enzymes CD39 and CD73 [10]. ATP generally acts as pro-inflammatory mediator by activation of purinergic P2 receptors [11] while adenosine signals through four different P1 purinergic receptors (A1, A2a, A2b, A3) mediating both anti- and pro-inflammatory effects depending on the receptor subtype [12].

Adenosine generated by CD39 and CD73 is well known to play a protective role in acute lung injury and lack of adenosine results in increased edema formation and prolonged inflammation [3]. The anti-inflammatory action of adenosine is predominantly mediated by the A2a receptor [13], [14] but also the A2b receptor appears to play a role in dampening ALI [8], [15]. First evidence that T lymphocytes considerably contribute to the resolution of ALI came from D'Alessio et al. showing a regulatory T cell-mediated cross-talk between innate and adaptive immune system that regulates the inflammatory environment in the lung after injury [16]. A recent study extended these findings by demonstrating that CD73-dependent adenosine generation by regulatory T cells may represent the key factor in the healing process [17]. We have reported that T cells display a high abundance of CD73 and that granulocytes and T cells infiltrating the injured heart after ischemia/reperfusion showed a significant upregulation of CD73 suggesting enhanced local formation of adenosine [18]. Deficiency of CD73 on immune cells and thus the lack of CD73-generated adenosine was associated with delayed resolution of inflammation and adverse remodeling [19]. We have also shown that CD73-derived adenosine tonically inhibits active NF-κB in regulatory T and effector T cells, thereby modulating the release of a broad spectrum of pro-inflammatory cytokines and chemokines [20]. Since T lymphocytes orchestrate inflammatory responses and contribute to the resolution of inflammation [16], [17] this suggests a crucial role of T cell subsets and in particular adenosine in the healing process of ALI. NAD and cAMP, aside of ATP, were recently suggested as additional endogenous sources of adenosine [21], [22]. The quantitative contribution of distinct pathways to the adenosine *in vivo* is currently unknown.

Since a detailed evaluation of purinergic signaling on infiltrating T cells during ALI is currently lacking, we examined the expression profile of purinergic signaling molecules including various ecto-enzymes, transporters, channels and receptors in helper T cells, cytotoxic and regulatory T cells, during the resolution phase of lipopolysaccharide (LPS)-induced lung injury. We also explored the capacity of activated CD4^+^ T cells to enzymatically degrade ATP, NAD, cAMP to AMP and adenosine.

## Materials and Methods

### Animals and murine model of LPS-induced ALI

Animal experiments were performed in accordance with the national guidelines on animal care and were approved by the Bezirksregierung Duesseldorf. The wild type female mice (C57BL/6, 20–23 g body weight, 8–12 weeks of age) used in this study were bred at the Tierversuchsanlage of Heinrich-Heine-University, Duesseldorf, Germany. They were fed with a standard chow diet and received tap water ad libitum. Experimental groups were matched in age and weight.

Lipopolysaccharide (LPS) from Salmonella enteriditis (Sigma, Taufkirchen, Germany) was dissolved at 1 mg/mL in sterile 0.9% saline. To induce acute pulmonary inflammation, mice were intubated with a sterile plastic catheter, anaesthetized by mechanical ventilation with 1.5% isoflurane at a rate of 150 strokes/min and challenged with intratracheal instillation of 60 µg of LPS dissolved in 60 µl of normal saline. After euthanasia by isoflurane inhalation blood, tissue and bronchoaveloar lavage (BAL) was collected under basal conditions and 3 d or 7 d after LPS treatment.

### Sample acquisition, tissue digestion and cell isolation

#### Blood

Blood was collected by cardiac puncture after injection of 2500 U heparin. For isolation of blood leukocytes whole blood was treated with ACK buffer (0.15 M NH_4_Cl, 1 mM KHCO_3_, 0.1 mM EDTA, pH 7.3) to lyse erythrocytes; thereafter cells were resuspended in MACS buffer (PBS, 0.5% BSA, 5 mM EDTA, pH 7.4).

#### Bronchoalveolar lavage

To obtain BAL fluid, the tracheal tube was disconnected from the mechanical ventilator after intubation and the lungs were lavaged 7 times with 1 ml of PBS. All removed fluid was centrifuged immediately for harvesting of the cells. Cells were finally resuspended in MACS buffer.

#### Lung tissue

Cells from the circulation were removed from the pulmonary vasculature by flushing the lung with 10 ml of PBS at 25 cmH_2_O through the spontaneously beating right ventricle. After removal of the heart and surrounding connective tissue, lungs were mechanically dissociated with a McIlwain tissue chopper (TC752) and gently dissociated by sequent pipetting steps, then digested for 1 hr at 37 °C in 10 ml PBS containing 10 mg collagenase II (295 U/mg, BioChrom AG, Berlin, Germany), 1 mg hyaluronidase (Sigma, Taufkirchen, Germany), and 300 U DNase I (Sigma, Taufkirchen, Germany). Digested lungs were filtered through a 70 µm BD Falcon cell strainer (BD Falcon, Bedford, MA, USA), the resulting cells were washed with PBS, and erythrocytes were lysed with ACK buffer. Centrifugation and resuspension of cells in MACS buffer followed.

### Antibodies and flow cytometry

Cells were resuspended in MACS buffer, preincubated with FcR Blocking Reagent (Miltenyi Biotech, Auburn, CA, USA), and DAPI-stained to exclude dead and apoptotic immune cells. Then, immune cells were stained with the following antibodies: APC-conjugated anti-mouse **CD3ε** (clone: 145-2C11; isotype: hamster IgG1), **CD11b** (clone: M1/70.15.11.5; isotype: rat IgG2b), **CD49b** (DX5) (clone: DX5; isotype: rat IgM), **Anti-NKp46** (clone: 29A1.4.9; isotype: rat IgG2a), and PE-conjugated anti-mouse **CD45** (clone 30F11.1; isotype: rat IgG2b) antibodies were purchased from **Miltenyi Biotec** (Auburn, CA, USA). APC-H7-conjugated anti-mouse **CD8a** (clone: 53–6.7; isotype: Rat (LOU) IgG2a, κ), APC-Cy7-conjugated anti-mouse **Ly-6C** (clone: AL-21; isotype: Rat IgM, κ), PerCP-Cy5.5-conjugated anti-mouse **CD4** (clone: RM4-5; isotype: Rat (DA) IgG2a, κ), **CD11c** (clone: HL3; isotype: Armenian Hamster IgG1, λ2), and PE-Cy7-conjugated anti-mouse **CD25** (clone: PC61; isotype: Rat (OFA) IgG1, λ) antibodies were purchased from **BD Bioscience** (Franklin Lakes, NJ, USA). APC-eFluor780-conjugated anti-mouse **F4/80** (clone: BM8; isotype: Rat IgG2a, κ), Anti-Human/Mouse **CD45R (B220)** (clone: RA3-6B2; isotype: Rat IgG2a, κ), PE-conjugated Anti-Mouse/Rat **Foxp3** (clone: FJK-16s; isotype: Rat IgG2a, κ), PerCP-Cy5.5-conjugated anti-mouse **Ly-6G (Gr-1)** (clone: RB6-8C5; isotype: Rat IgG2b, κ), **CD11c** (clone: HL3; isotype: Armenian Hamster IgG), PE-Cy7-conjugated anti-mouse **CD39** (clone: 24DMS1; isotype: Rat IgG2b, κ) and eFluor450-conjugated anti-mouse **CD45** (clone: 30-F11; isotype: Rat IgG2b, κ) antibodies were purchased from **eBioscience** (San Diego, CA, USA). FITC-conjugated anti-mouse **CD73** (clone: 496406; isotype: Rat IgG2a) antibody was purchased from **R&D Systems** (Minneapolis, MN, USA). Erythrocytes were stained with a PE-conjugated anti-mouse **TER-119** (clone: Ter119; isotype: Rat IgG2b, κ) from **BD Bioscience** (Franklin Lakes, NJ, USA).

To identify the individual subsets of the total pool of CD45^+^ immune cells we used a panel of antibodies against different cell-specific leukocyte markers: cytotoxic T cells (CD3^+^, CD8^+^), T helper cells (CD3^+^, CD4^+^), regulatory T cells (CD3^+^, CD4^+^, CD25^+^, FoxP3^+^), B cells (CD45R(B220)^+^), NK cells (CD49b(DX5)^+^, NKp46^+^), granulocytes (CD11b^+^, Ly6g^+^), macrophages and monocytes (CD11b^+^, Ly6g^−^, CD11c^−^, Ly6c^low/high^), alveolar macrophages (CD11b^−^, CD11c^+^) and myeloid antigen-presenting cells (APCs; CD11b^+^, Ly6g^−^, CD11c^+^, F4/80^−/+^, MHCII^−/+^). For intracellular staining of Foxp3, we used the Foxp3-Staining Buffer Set (eBioscience, San Diego, CA, USA) according to the manufacturer's protocol. To exclude a possible contamination of the lung tissue immune cells with immune cells from circulating blood we determined the number of erythrocytes in lung tissue after flushing and enzymatic digestion. Assuming the ratio of erythrocytes to leukocytes in peripheral blood to be 1000:1, contamination with blood derived leukocytes was calculated to be less than 0.2% (n = 5).

Cell measurements were performed with a FACSCanto II flow cytometer (BD Bioscience, Heidelberg, Germany). For analysis, the placement of gates was based on fluorescence minus one (FMO) controls. The minimum number of events used to define a cell population was 150. The analysis was always performed on individual lungs. The different immune cells subsets were enumerated and the percentage of CD39 and CD73 expressing cells and corresponding expression levels as measured by the mean fluorescence intensity (MFI) was assessed.

### Isolation of T helper cells, cytotoxic and regulatory T cells

Total immune cells were isolated from the lung tissue 7 d after LPS challenge and T cells were stained as described above. The T helper, cytotoxic T and regulatory T cells were extracted from the total immune cells by fluorescent activated cell sorting (FACS) on a MoFlo XDP cell sorter (Beckman Coulter, CA, USA). The sort criteria were as follows: non-viable cells were excluded on the basis of a positive DAPI staining. Next, doublets were excluded on the basis of the width of their 90° scatter signal. The sort gates for the three T cell subsets were finally set according to the staining for T helper cells (CD45+, CD3+, CD4+), cytotoxic T cells (CD45+, CD3+, CD8+) and regulatory T cells (CD45+, CD3+, CD4+, CD25+). In separate experiments (n = 18) purity of the sorted populations was assessed and was about 93.6+/−4.3%.

### Quantitative *real-time* PCR

Quantitative *real-time* PCR was performed to determine LPS-induced changes in the mRNA expression of nucleotide and nucleoside degrading enzymes, receptors, transporters and channels in T cell subsets from whole lung tissue (Table S1). After sorting of T cell subsets total RNA was isolated using the RNeasy Micro Kit and cDNA was synthesized applying the QuantiTect Reverse Transcription Kit (Qiagen GmbH, Hilden, Germany) according to the manufacturer's instructions.

Quantitative *real-time* PCR was conducted using preloaded TaqMan Array Microfluidic Cards and the ViiA 7 System (Applied Biosystems, Darmstadt, Germany) following the manufacturer's protocol and all PCR assays were performed in triplicate. Gene expression was normalized to beta-actin. Fold changes in gene expression were calculated by comparing expression levels in T cells from the unstressed lung to that from the injured lung 7 d post injury. When mRNA expression was below the detection limit, fold changes could not be calculated and the relative expression levels are depicted.

### HPLC analysis of nucleotide and nucleoside metabolism

CD4^+^ T cells isolated from the injured lungs 7 d post induction of ALI were sorted into GIBCO Hank's Balanced Salt Solution (HBSS) using FACS. After two washing steps with HBSS 250.000 cells were resuspended in 350 µl HBSS. The nucleotides ATP, AMP, cAMP and ADP-ribose (ADPR) were then added with a final concentration of 20 µM and incubated at 37 °C. To stop the reaction at indicated time points (2, 4, 12, 30 and 60 min) an aliquot of 60 µl of the incubation medium was transferred into an Eppendorf tube on ice and centrifuged at 4 °C for 2.5 min at 550 g. Aliquots of 30 µl were applied to a reverse phase HPLC system using a Hypersil BDS C18 column (Thermo Fisher Scientific, Langerwehe, Germany) as previously described [23]. Briefly, purines were separated by running a linear gradient of buffer A (150 mM potassium dihydrogen orthophosphate) and buffer B (15% acetonitrile in buffer A) using a low pressure gradient mixing device. The absorbance was measured at 254 nm and the retention times were assessed using standard samples of nucleotides and nucleosides.

### Statistics

Data are represented as mean ± SD. When comparing the means of more than two groups significances were calculated using one-way ANOVA with Dunnett's post hoc test. For the comparison of two means the Mann-Whitney U test was applied. Statistical analyses were performed using Graph Pad Prism 4 (GraphPad Software Inc., San Diego, CA, USA) and the threshold for statistical significance was set at *P*<0.05.

## Results

### CD39 and CD73 expression on leukocyte subpopulations after LPS challenge

**Figure 1:**
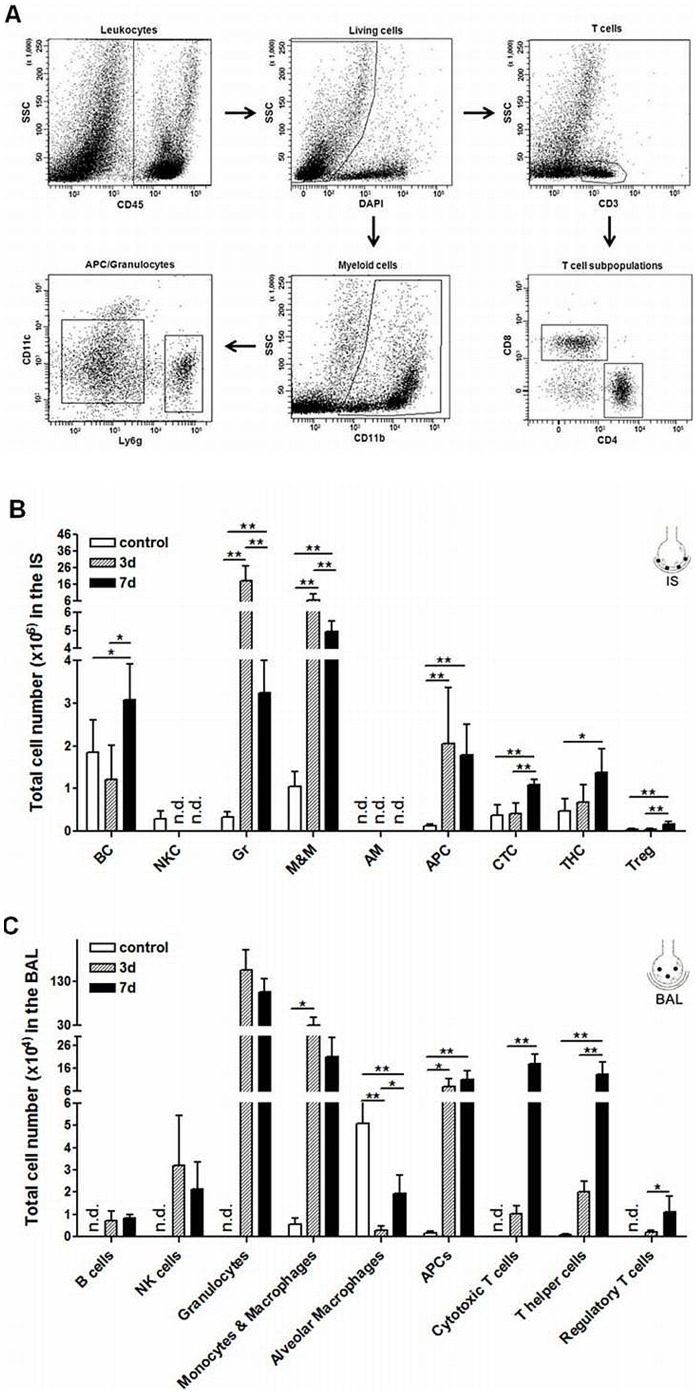
Dynamic changes of distinct leukocyte subpopulations in lung tissue (IS) and bronchoalveolar lavage (BAL) 3 d and 7 d after intratracheal instillation of LPS. (A) Representative plots illustrating the gating strategy used to identify different leukocyte subpopulations by flow cytometry. Non-viable immune cells were excluded from the CD45^+^ cells on the basis of a positive DAPI-staining. Viable CD45^+^ cells were then divided into subpopulations using a panel of cell-specific fluorochrome-labeled antibodies. Lymphocytes were gated for CD45R(B220)^+^ cells (B cells) and CD3^+^ cells (T cells). T cells were subdivided into CD4^+^ cells (T helper cells) and CD8^+^ cells (cytotoxic T cells). Myeloid cells were defined as CD11b^+^ cells and further subdivided into CD11c^+^ cells (APCs) and Ly6g^+^ cells (granulocytes) (B) In IS the early phase of inflammation (3 d) was characterized by a transient increase in myeloid immune cell subsets. In the later phase of inflammation (7 d), different T cell subsets were significantly enhanced. (C) Similar to the lung tissue, myeloid immune cell subsets were increased in BAL in the early phase while T cells were enhanced only in the later phase of inflammation. Data are mean ± SD (n = 5 mice per group) Statistical significance was assessed by one-way ANOVA with Dunnett's post hoc test. *P<0.05, **P<0.01, ***P<0.0001. ALI  =  acute lung injury, AM  =  alveolar macrophages, APC  =  antigen-presenting cells, BAL  =  bronchoalveolar lavage, BC  =  B cells, CTC  =  cytotoxic T cells, Gr  =  granulocytes, IS  =  interstitial lung tissue, LPS  =  lipopolysaccharide, M&M  =  monocytes and macrophages, n.d.  =  not detected, NKC  =  natural killer cells, SD  =  standard deviation, THC  =  T helper cells, Treg  =  regulatory T cells.

Leukocyte subsets in lung tissue (IS), bronchoalveolar lavage (BAL) and intravascular space (IV) were measured under control conditions and 3 d and 7 d after LPS-induced lung injury. Representative plots illustrating the gates that were set to enumerate the immune cell subpopulations are shown in Figure 1A. Under unstressed conditions, lymphocytes represent the dominant cell type in the lung tissue followed by monocytes and macrophages, granulocytes and natural killer (NK) cells (Figure 1B). A similar pattern was observed in the intravascular compartment (Figure S1A). In BAL, alveolar macrophages were the most prominent cell fraction while lymphocytes and other myeloid cell subsets were less abundant or could not be detected (Figure 1C). The early phase of inflammation (3d after LPS instillation) was characterized by considerably increased numbers of granulocytes (67-fold, P<0.001), monocytes and macrophages (7.7-fold, P<0.001) and antigen-presenting cells (APCs) (20-fold, P<0.001) in lung tissue. This increase was transient and after 7 d the respective changes in cell numbers were still about 10-fold (P<0.001), 5-fold (P<0.001) and 15-fold (P<0.01) above baseline. The later phase of the inflammatory process (7 d after LPS exposure) was characterized by an elevated number of all T cell subsets (cytotoxic T cells: 2.5-fold increase, P<0.001; T helper cells: 2.4-fold increase, P<0.05; regulatory T cells: 3.7-fold increase, P<0.01) (Figure 1B). Similar dynamic changes of leukocyte subsets were observed in BAL (Figure 1C). Note, that in the intravascular compartment B cells, NK cells and T cells transiently decreased 3 d and re-increased 7 d after LPS exposure while granulocytes remained elevated over the period of observation (Figure S1A).

**Figure 2:**
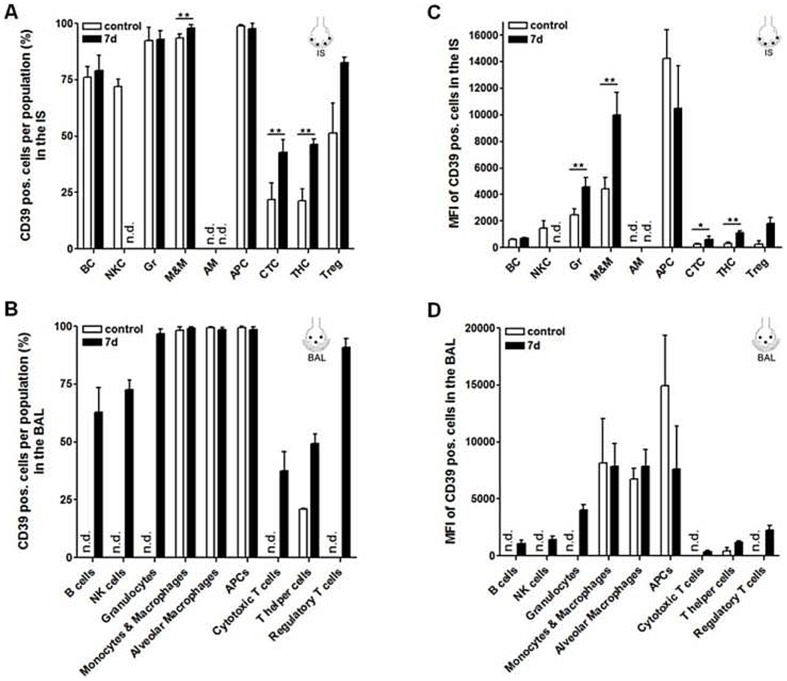
Percentage of CD39 positive cells within and expression pattern (MFI) of CD39 on the different leukocyte subpopulations 7 d post induction of ALI analyzed by flow cytometry. (A+B) The percentage of cells expressing CD39 was high within the myeloid cell, B cell and NK cell populations but low within the different T cell subsets and was increased within the T cell subsets after ALI. (C+D) As assessed by means of the MFI CD39 was highly expressed on myeloid cells, B cells and NK cells and showed a comparatively low expression on cytotoxic and T helper cells. After LPS installation, aside from Gr and M&M, particular T cells showed an increased abundance of CD39. Data are mean ± SD (n = 5 mice per group). Statistical significance was assessed by one-way ANOVA with Dunnett's post hoc test. *P<0.05, **P<0.01, ***P<0.0001. Under unstressed conditions CD39 staining of regulatory T cells (IS) and T helper cells (BAL) was detected in only n = 2, thus statistical significance was not assessed. ALI  =  acute lung injury, AM  =  alveolar macrophages, APC  =  antigen-presenting cells, BAL  =  bronchoalveolar lavage, BC  =  B cells, CTC  =  cytotoxic T cells, Gr  =  granulocytes, IS  =  interstitial lung tissue, MFI  =  mean fluorescence intensity, M&M  =  monocytes and macrophages, n.d.  =  not detected, NKC  =  natural killer cells, SD  =  standard deviation, THC  =  T helper cells, Treg  =  regulatory T cells.

**Figure 3:**
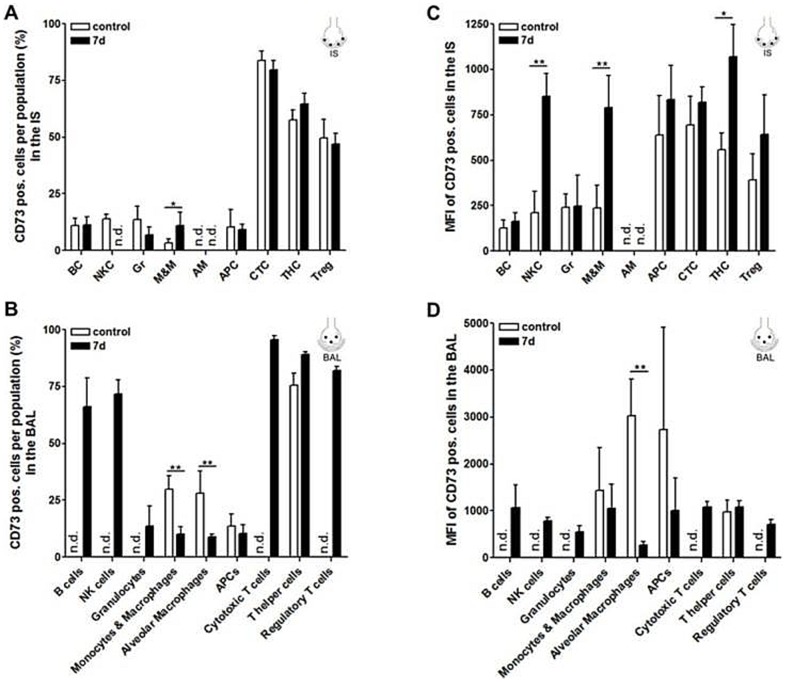
Percentage of CD73 positive cells within and expression pattern (MFI) of CD73 on the different leukocyte subpopulations 7 d post induction of ALI analyzed by flow cytometry. (A+B) The percentage of cells expressing CD73 was high within the T cell subsets but low within the myeloid cell, B cell and NK cell populations. The percentage of CD73 expressing cells tended to be increased in T helper cells after ALI. (C+D) As assessed by means of the MFI CD73 was highly expressed on the different T cell subsets and showed a comparatively low expression on myeloid cells, B cells and NK cells. After LPS installation particular T helper cells, NKC and M&M showed an increased abundance of CD73. Data are mean ± SD (n = 5 mice per group). Statistical significance was assessed by one-way ANOVA with Dunnett's post hoc test. *P<0.05, **P<0.01, ***P<0.0001. Under unstressed conditions CD73 staining of regulatory T cells (IS) and T helper cells (BAL) was detected in only n = 2, thus statistical significance was not assessed. ALI  =  acute lung injury, AM  =  alveolar macrophages, APC  =  antigen-presenting cells, BAL  =  bronchoalveolar lavage, BC  =  B cells, CTC  =  cytotoxic T cells, Gr  =  granulocytes, IS  =  interstitial lung tissue, MFI  =  mean fluorescence intensity, M&M  =  monocytes and macrophages, n.d.  =  not detected, NKC  =  natural killer cells, SD  =  standard deviation, THC  =  T helper cells, Treg  =  regulatory T cells.

We next examined the abundance of CD39 and CD73 within the different immune cell subsets. As summarized in Figure 2A, the percentage of cells expressing CD39 in IS was high within myeloid cell, B cell and NK cell populations but low within the different T cell subsets. In contrast, CD73 expressing cells were highly abundant within all T cell subsets but less present within myeloid cell, B cell and NK cell populations (Figure 3A). A similar pattern was observed within the cell populations analyzed from the intravascular space (Figure S2A+B). Analysis of the immune cells from BAL revealed that CD39 expressing cells were highly abundant within the alveolar macrophage, APC and monocyte and macrophage populations but were less abundant within the T helper and cytotoxic T cell subset which again displayed a high percentage of cells with expression of CD73 (Figure 2B+3B). Similar to the percentage of CD39 and CD73 positive cells, the expression levels of CD39 were also higher on myeloid cells but lower on lymphoid cells in all three compartments while the opposite was true for CD73 (Figure 2C+D, Figure 3C+D and Figure S2C+D). At day 7 after LPS challenge, the amount of cells positive for CD39 and CD73 as well as the expression level of those molecules was increased particularly in the T cell subsets in both, the lung tissue and BAL (Figure 2A–D and 3A–D). We observed an increase of similar magnitude already on day 3 (data not shown). Representative histograms of the flow cytometric analysis for CD39 and CD73 expression as well as FMO controls are shown in Figure S1B+C.

**Figure 4:**
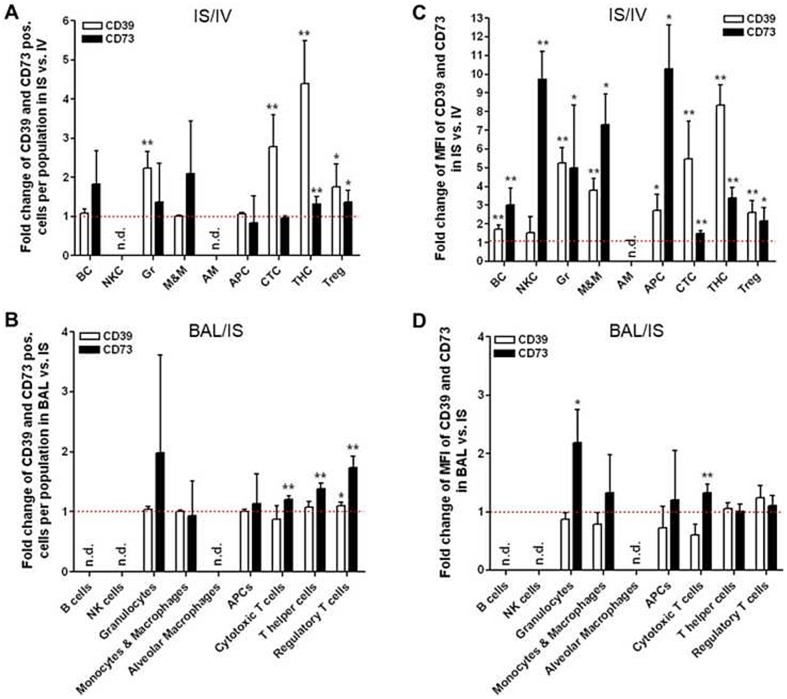
Ratios ( =  fold changes) of CD39 and CD73 expression (percentage and MFI) on immune cell subsets in IS compared to IV (IS/IV) and in BAL compared to IS (BAL/IS) 7 d post induction of ALI. (A+B) Portion of CD39 and CD73 expressing cells is particularly increased within the T cell subsets from IS compared to that from IV 7 d post induction of ALI. Analysis of the ratio BAL/IS revealed that CD73 expressing cells were significantly augmented within the T cells subsets while changes in CD39 positive cells were small. (C+D) Expression levels of CD39 and CD73 assessed by MFI were significantly upregulated on nearly all immune cell subsets from IS compared to that from IV 7 d post induction of ALI. A significant increased expression of CD73 was found on granulocytes and cytotoxic T cell when analyzing the ratio BAL/IS. Data are mean ± SD (n = 5 mice per group). Ratios were calculated by dividing the fraction of positive cells or MFI of immune cell subsets in IS by that in IV (IS/IV) or in BAL by that in IS (BAL/IS) 7 d after induction of ALI. Statistical significance was assessed by Mann-Whitney U test. *P<0.05, **P<0.01, ***P<0.0001. ALI  =  acute lung injury, AM  =  alveolar macrophages, APC  =  antigen-presenting cells, BAL  =  bronchoalveolar lavage, BC  =  B cells, CTC  =  cytotoxic T cells, Gr  =  granulocytes, IS  =  interstitial lung tissue, IV  =  intravascular space, MFI  =  mean fluorescence intensity, M&M  =  monocytes and macrophages, n.d.  =  not detected, NKC  =  natural killer cells, SD  =  standard deviation, THC  =  T helper cells, Treg  =  regulatory T cells.

To further analyze the changes in CD39 and CD73 abundance on the different immune cell subsets during migration from IV to IS and finally to BAL, we calculated the ratios ( =  fold change) of IS/IV and BAL/IS for CD39 and CD73 positive cells and for corresponding expression levels 7 d after induction of ALI. As shown in Figure 4A, analysis of the IS/IV ratio revealed that the percentage of CD39 expressing cells was increased within granulocyte (2.2-fold, P<0.01), cytotoxic T cell (2.8-fold, P<0.01), T helper cell (4.4-fold, P<0.01) and regulatory T cell subsets (1.8-fold, P<0.05). On the other hand, CD73 abundance was significantly elevated within T helper cell (1.3-fold, P<0.01) and regulatory T cell subsets (1.4-fold, P<0.05). Similar directional changes were also observed when analyzing the ratio BAL/IS (Figure 4B): CD73 expressing cells were significantly augmented within cytotoxic T cell (1.2-fold, P<0.01), T helper cell (1.4-fold, P<0.01), and regulatory T cell subsets (1.7-fold, P<0.01) while changes in CD39 positive cells were small and reached the level of significance only within regulatory T cell subset (1.1-fold, P<0.05). The respective fold changes of expression levels as a measure for the migration from blood to lung tissue (IS/IV) and from lung tissue to bronchoalveolar lavage (BAL/IS) are depicted in Figure 4C+D. Similar to changes of CD39 and CD73 expressing cells within the T cell populations, we found increased expression levels of both ectoenzymes on infiltrating T cell subsets. Analysis of the IS/IV ratio showed a significantly enhanced expression of CD39 on cytotoxic T cells (5.5-fold, P<0.01), T helper cells (8.3-fold, P<0.01) and regulatory T cells (2.6-fold, P<0.01). Similarly, CD73 was significantly increased on cytotoxic T cells (1.5-fold, P<0.01), T helper cells (3.4-fold, P<0.01), and regulatory T cells (2.1-fold, P<0.05). Calculation of the BAL/IS ratio revealed an augmented expression of CD73 on cytotoxic T cells (1.3-fold, P<0.01).

### Expression profile of purinergic enzymes, transporter, channels and receptors within T cell subsets

The increased surface expression of CD39 and CD73 after LPS exposure particularly on T cells prompted us to examine the expression profile of various ectoenzymes involved in extracellular ATP and NAD degradation including all relevant P1 and P2 receptors at the mRNA level. For this purpose, we sorted T helper, cytotoxic and regulatory T cells using FACS 7 d after LPS exposure and performed *real-time* PCR (see list in Table S1). Note, that CD3+, CD4+ and CD25+ T cells can include both regulatory T cells and activated T cells.

**Figure 5:**
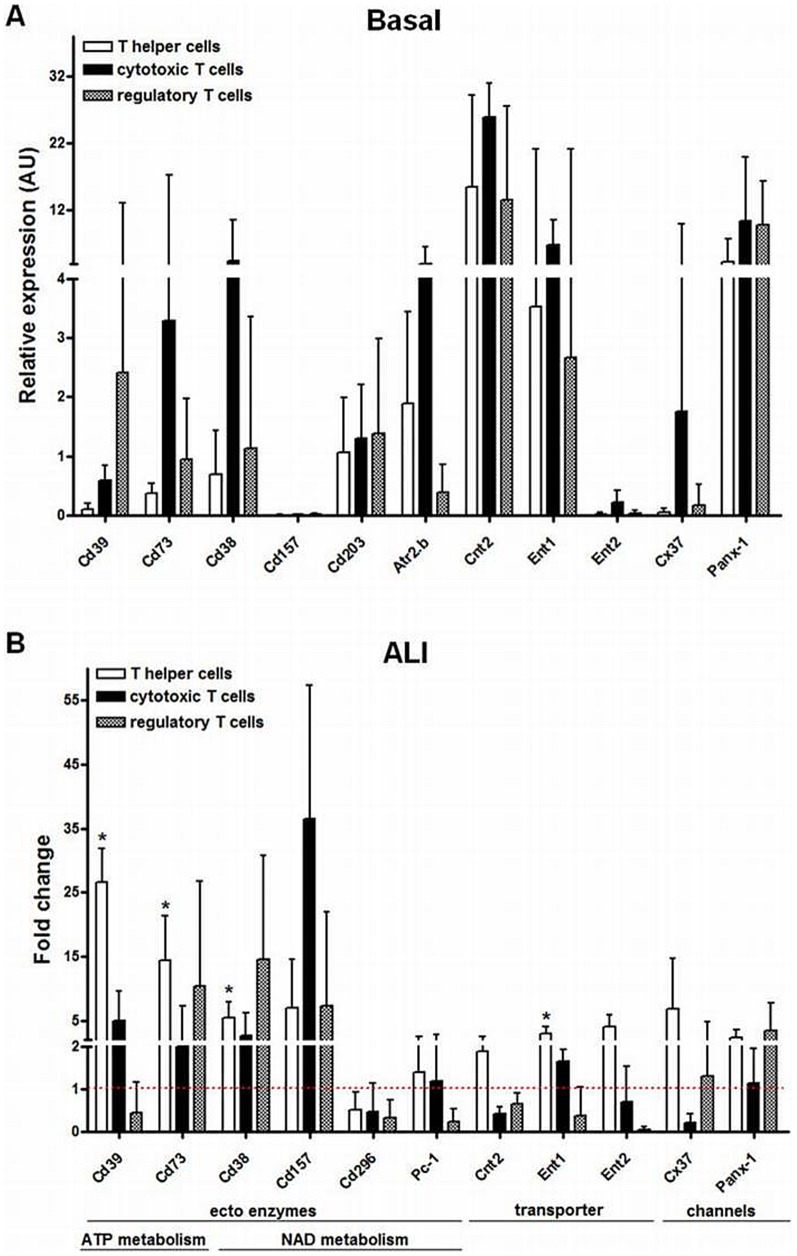
Gene expression of distinct ectoenzymes, transporters and channels in the T cell subsets isolated from lung tissue under basal conditions and 7*real-time* PCR. (A) Under basal conditions, T cell subsets expressed various ectoenzymes of ATP and NAD degradation cascade as well as nucleotide and nucleoside transporters and channels. Gene expression was normalized to beta-actin and relative expression levels are depicted. (B) In the diseased state, *Cd38*, *Cd39* and *Cd73* were significantly upregulated on T helper cells and tended to be increased in cytotoxic and regulatory T cells. *Ent1* transcripts were significantly increased in T helper cells. Data are mean ± SD (n = 4 mice per group). Fold changes were calculated by comparing the expression under basal conditions to that in the injured lung 7 d post induction of ALI and statistical significance was then assessed by Mann-Whitney U test. *P<0.05, **P<0.01, ***P<0.0001. ALI  =  acute lung injury, Art2.b  =  ADP-ribosyltransferase 2b, ATP  =  adenosine triphosphate, AU  =  arbitrary units, Cnt  =  concentrative nucleoside transporter, Cx  =  connexin, Ent  =  equilibrative nucleoside transporter, LPS  =  lipopolysacch, NAD  =  nicotinamide adenine dinucleotide, Panx-1  =  pannexin-1, SD  =  standard deviation.

As shown in Figure 5A, highest expression values in all T cell subsets under basal conditions were found for the *concentrative nucleoside transporter 2* (*Cnt2*), *equilibrative nucleoside transporter* 1 (*Ent1*) and *Pannexin 1* (*Panx-1*). T cell subsets expressed *Cd38* which degrades NAD. The other NAD metabolizing enzyme *Cd157* was of very low abundance and *Cd203* was below detection limit (Figure S3A). Among the ATP degrading enzymes, regulatory T cells show the highest *Cd39* expression while expression of *Cd73* was highest in cytotoxic T cells. Similar to data shown in Figure 2C and 3C, ALI upregulated *Cd39* and *Cd73* in T helper cells about 27-fold (P<0.05) and 14-fold (P<0.05), respectively. Alkaline phosphatase *Alp* which also can break down adenine phosphates was found not to be expressed in T cells (Figure S3). The increase in *Cd38* was about 5-fold (P<0.05). None of the other measured transporters and channels reached the level of significance except the 3.0-fold increase (P<0.05) in *Ent1* on T helper cells (Figure 5B). The expression of further adenosine degrading enzymes such as *adenosine deaminase* (*Ada*) and *adenosine kinase* (*Adk*) showed no significant changes upon ALI in T cell subsets (Figure S3B).

**Figure 6:**
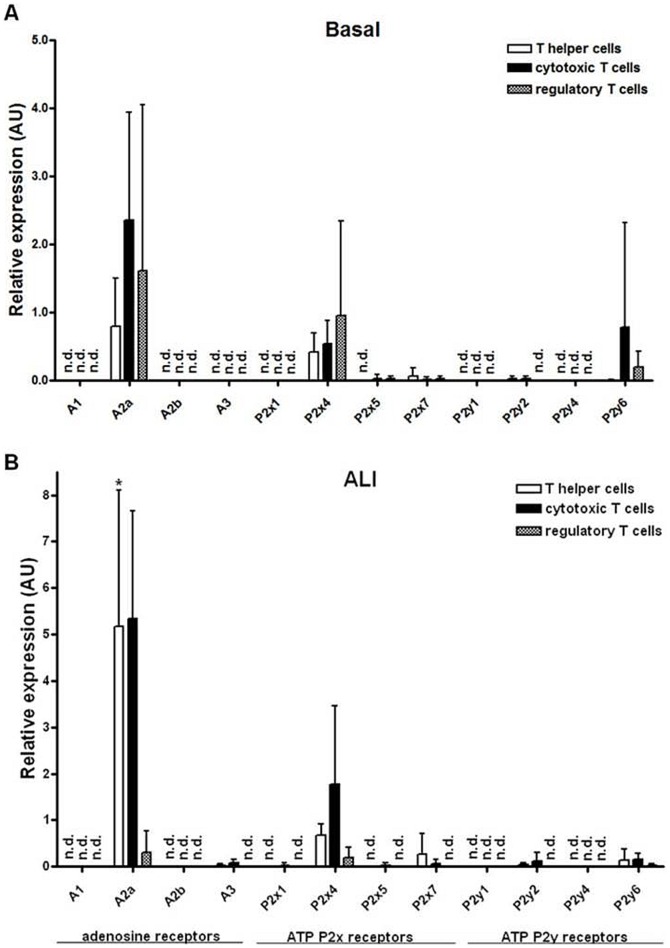
Gene expression of adenosine and ATP receptors in the T cell subsets isolated from the lung under basal conditions and 7d after LPS exposure determined by quantitative *real-time* PCR. (A) In the unstressed lung, T cells expressed predominantly the *A2a receptor*. The *P2x4* and *P2y6 receptors* were moderately expressed while *P2x5/7* and *P2y2 receptors* displayed a very low expression level. Gene expression was normalized to beta-actin and relative expression levels are depicted. (B) LPS administration significantly induced the *A2a receptor* expression in T helper cells 7 d post instillation. Since expression of some target mRNAs was below the detection limit in at least one of the conditions, fold changes (control vs. 7 d) were not calculated but the relative expression levels were depicted. Data are mean ± SD (n = 4 mice per group). Expression levels under basal conditions were compared to that in the injured lung 7 d post induction of ALI and statistical significance was then assessed by Mann-Whitney U test. *P<0.05, **P<0.01, ***P<0.0001. ALI  =  acute lung injury, ATP  =  adenosine triphosphate, AU  =  arbitrary units, LPS  =  lipopolysaccharide, n.d.  =  not detected, SD  =  standard deviation.

Figure 6A shows that in the unstressed lung the *A2a receptor* is the dominant adenosine receptor on all T cell subsets. Among the ATP receptors studied only the *P2x4* and *P2y6 receptors* were moderately expressed. ALI significantly increased the *A2a receptor* expression in T helper cells (6.5-fold, P<0.05) and also by trend in cytotoxic T cells (Figure 6B). Note, that the *A3 receptor* expression was above the detection threshold in T helper and cytotoxic T cells isolated from the diseased lung 7d after LPS administration.

### Metabolism of extracellular nucleotides by activated CD4^+^ T cells

**Figure 7:**
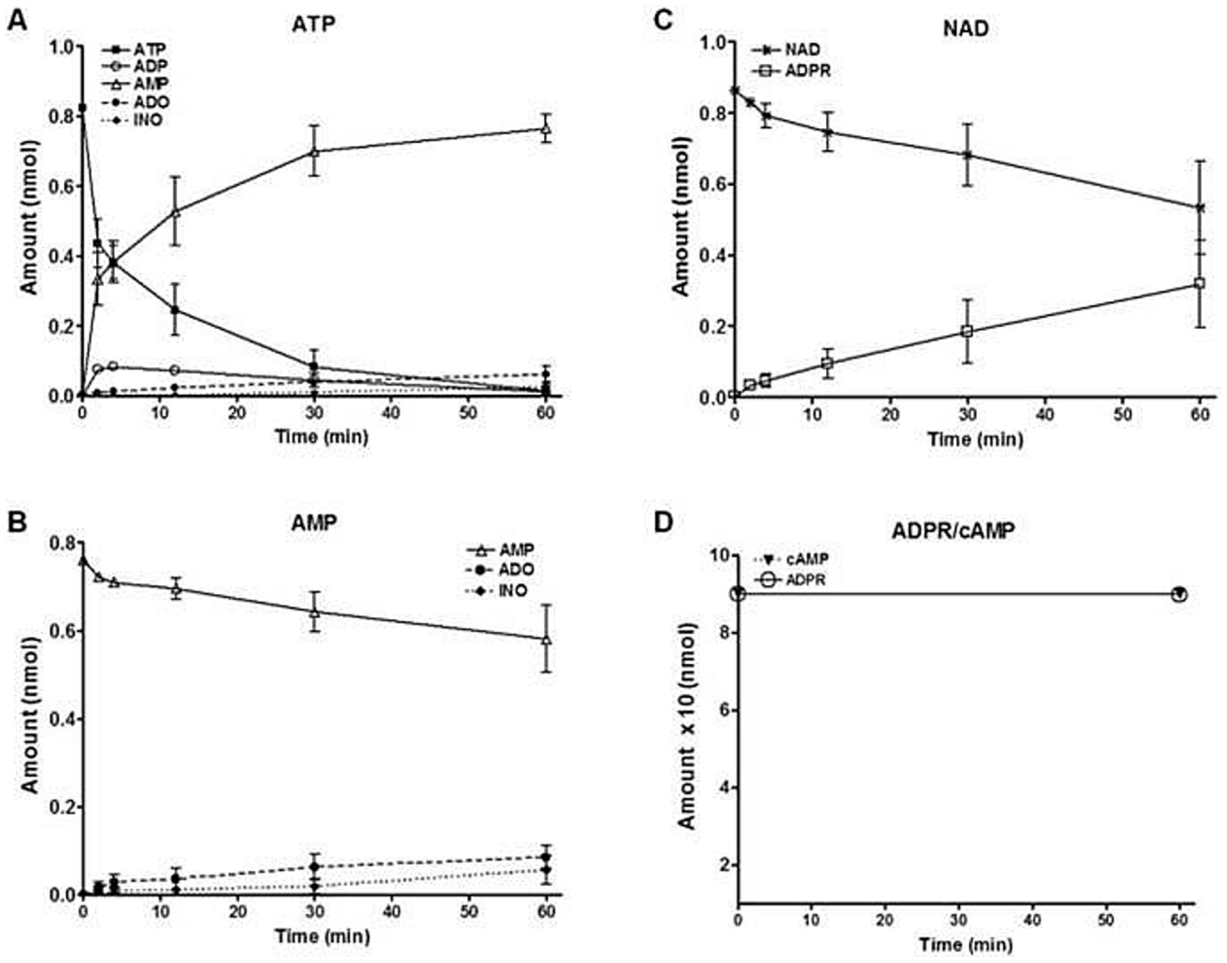
Extracellular degradation of ATP, AMP, NAD, cAMP, ADPR (substrate concentration: 20 µM; 37°C) by activated CD4^+^ T cells isolated from the lung 7 d post induction of ALI. (A) Rapid ATP degradation to ADP and AMP and slow adenosine formation. (B) AMP only was added as substrate was slowly degraded with concomitant formation of adenosine and inosine. (C) NAD is degraded to ADPR at a moderate rate. (D) CD4^+^ T cells do not degrade ADPR and cAMP. ADO  =  adenosine, ADP  =  adenosine diphosphate, ADPR  =  adenosine diphosphate ribose, ALI  =  acute lung injury, AMP  =  adenosine monophosphate, ATP  =  adenosine triphosphate, cAMP  =  cyclic adenosine monophosphate, INO  =  inosine, NAD  =  nicotinamide adenine dinucleotide.

Since *Cd38*, *Cd39* and *Cd73* were significantly upregulated in CD4^+^ T helper cells isolated from the injured lungs, we assessed the functional relevance of these findings by measuring the extracellular degradation of ATP, AMP, cAMP, NAD and ADPR (20 µM each) by activated CD4^+^ T cells using HPLC. CD4^+^ T cells were isolated from the lung 7 d post induction of ALI and the kinetics were measured over 60 min. As shown in Figure 7A, ATP is rapidly degraded, ADP transiently increased and AMP appeared as the main end product with little adenosine formation. This observation was confirmed by the finding that AMP is only slowly degraded with concomitant formation of adenosine and inosine (Figure 7B). As can be seen in Figure 7C, NAD is metabolized to ADPR at a comparatively moderate rate and no other degradation products were observed. Consistent with this latter finding, ADPR was not measurably degraded over 60 min. Similarly, when cAMP was used as substrate no degradation was measureable (Figure 7D).

## Discussion

This study explored dynamic changes of purinergic signaling molecules on leukocytes, particular T cells, infiltrating the lung after ALI. We found that the transcriptional phenotype of T helper cells, cytotoxic and regulatory T cells displayed increased ATP and NAD catabolism. However, measurement of enzyme activities revealed that only ATP but not NAD is a source for the generation of AMP and adenosine in intact cells. Furthermore, the A2a receptor is the dominant receptor subtype on all T cell subsets and becomes upregulated after ALI. This suggests that the CD73-A2a-receptor axis on T cells plays an important role in the healing process.

ALI is characterized by a considerable influx of myeloid cells into the lung tissue and the bronchoalveolar compartment in the early phase and of T cells in the later phase of inflammation [5], [24]. The present study confirmed this observation. Consistent with previous data in the heart [18], we also found CD39 to be highly expressed on myeloid cells and shows only low expression on lymphoid cells, while the opposite is true for CD73. The reasons for the different contribution of immune cells to ATP degradation is presently unclear, but may relate to metabolic compartmentation of immune cell subtypes [19]. The interplay between the different leukocyte subsets with respect to purinergic signaling deserves further attention and may be important to shape the immune response.

We found that LPS-induced lung injury enhanced the abundance of CD39 and CD73 on all three T cell subsets infiltrating the lung tissue and the bronchoalveolar space. There appears to be a gradual increase in the abundance of the ectoenzymes when immune cells migrate from the circulation to the lung tissue and finally to the bronchoalveloar space. This most likely reflects changes in the microenvironments the cells are exposed to and is likely to be multifactorial. It has been reported that CD39 and CD73 are upregulated by hypoxia [25]–[27], which occurs during ALI or may be the direct result of hypoxic gene activation by LPS [28]. It is also conceivable, that infiltrating immune cells display an activated phenotype associated with increased secretion of pro-inflammatory cytokines [29] which secondarily may lead to the upregulation of the ectoenzymes. Interestingly, T cells subsets from the bronchoalvelar space showed the highest abundance of CD73. T cells from BAL of the uninjured rat lung have already been reported to exhibit an increased expression of activation markers such as IL-2R and ICAM-1 [30]. Also, the presence of activated alveolar macrophages in the bronchoalveolar space may have contributed to the upregulation of CD39 and CD73 abundance. Note, that regulatory T cells were shown to interact with alveolar macrophages resulting in decreased production of pro-inflammatory cytokines [16], which may be adenosine related. Thus, T cells appear to adapt to and subsequently modulate the local inflammatory microenvironment in different lung compartments by upregulation of CD39 and CD73.

T cells have been reported to release ATP [31] which involves ATP channels such as connexin 37 and 43 as well as pannexin-1 [31], [32]. We found that neither *connexins* nor *pannexin-1* were transcriptionally induced in T cells after ALI suggesting that these T cells rely on other cellular sources of ATP for the further degradation to adenosine. In addition, the *P2x7 receptor*, reported to enhance ATP release from T cells [31], was only marginally expressed and was not modulated by ALI. Therefore, irrespectively of its original source, the adenosine formed by T cells is well known to inhibit the pro-inflammatory cytokine production [20] and to influence the activation status of lung tissue macrophages, alveolar macrophages, neutrophils and possibly B cells, which all express adenosine receptors depending on their activation state [14]. This paracrine, adenosine-mediated purinergic response is likely to favor the resolution of the pulmonary inflammation [6] orchestrated by T cells.

It has been repeatedly reported that the P2X7 receptor is dominantly expressed on T cells and that autocrine activation leads to production of pro-inflammatory cytokines and proliferation [31], [33]. This does not pertain to T cells infiltrating the injured lung. Lung T cells show a rather modest expression of *P2x4* and the *P2y6 receptors* and only very low *P2x7 receptor* expression and none of these receptors is significantly modulated during ALI. P2X4 and P2Y6 receptors have been reported to mediate T cell activation [34], [35] and to be involved in ATP-induced chemotaxis [35], [36]. Others have also suggested that P2Y6 receptors are endogenous suppressors in the T cell driven pathogenesis of allergic pulmonary inflammation [37] and inhibit P2X4 function [38]. Given the only moderate expression of P2 receptors on lung T cells, together with the high activity of ATP degrading enzymes, this suggests that the biological half-life of ATP is rather short and does not permit significant activation of P2 receptors. This implies that P2X7-mediated apoptosis of T cells [33] may be negligible as long as high CD39 and CD73 activity is continuously present. Thus, the equilibrium between P2 and P1 activation is rather shifted to the dephosphorylated state with adenosine and with enhanced A2a receptor expression.

We found that *Cnt2* and *Ent1* transcript levels were highly expressed in T cells within the unstressed lung suggesting high basal adenosine transport between T cells and the microenvironment. The downregulation of nucleoside transporters has been hypothesized to enhance extracellular adenosine levels resulting in augmented adenosine receptor activation [39]. Conversely, in the present study, the high expression of *Ent1* and *Cnt2* rather suggests an increased uptake of extracellular adenosine which can terminate the adenosine receptor signaling [10]. Since high levels of extracellular adenosine are lymphotoxic [40], the continued expression of *Cnts* and *Ents* may reduce extracellular adenosine thereby counteracting possible detrimental effects of high extracellular adenosine.

In the literature the A2a and the A2b receptors were suggested to mediate the anti-inflammatory effect of adenosine in ALI [6], [8], [10]. In the present study, *A2b receptor* expression on T cell subsets was not detectable in the LPS-injured lung. We found the *A2a receptor* to be the dominant subtype which is well expressed under basal conditions and becomes even upregulated in T cells from the LPS-injured lung. This suggests that the CD73- A2a axis reflects the dominant anti-inflammatory signaling cascade on T cells infiltrating the injured lung that is known to attenuate inflammation and tissue damage [6].

Extracellular ATP generated during LPS-induced lung injury is likely to come from different sources. The initial tissue damage goes along with cell necrosis and apoptosis which is known to release intracellular ATP in high concentrations, however, only over a limited period of time. In a later phase when dead cells are removed and healing is initiated by the influx of immune cells, ATP is likely to be released from the activated immune cells themselves [9]. Upregulated CD73 together with increased A2a receptor and accelerated ATP catabolism via the CD73-A2aR axis on T cells may therefore be important in the resolution phase after lung injury and may serve as potential target for improving or accelerating the healing process. Note, that T cells did not express alkaline phosphatase, so that adenosine is exclusively produced by CD73.

Previous publications describe CD39 and CD73 as specific surface markers of regulatory T cells when isolated from lymph nodes and spleen [41], [42]. In the present study we show that CD39 and CD73 are robustly expressed on all three T cell subsets from blood, lung tissue and bronchoalveolar space and therefore contribute to the formation of adenosine. Moreover, others have reported that all 4 adenosine receptors were expressed in mouse T cells from lymph nodes or spleen [20], [43]. These discrepancies suggest that the microenvironment and maturation state of T cells may be critical for the expression of purinergic receptors and the ectonucleotide cascade. In line with this interpretation, maturation and activation state of T cells was reported to influence ectoenzyme expression levels [26], [27].

We found that ATPase activity is substantially higher then AMPase activity on T cells while flow cytometry analysis revealed a lower abundance of CD39 compared to CD73. A possible explanation could be that aside of CD39 there are additional ATPases expressed on T cells. This could include different NTPDases and pyrophosphateses on the cell surface [44]. Therefore CD39 may only be a part of the total active ectoATPase measured by HPLC techniques. In contrast CD73 can be equated with AMPase, since T cells are devoid of alkaline phosphates, the only other AMP degrading enzyme.

Measurement of ectoenzyme activities revealed that T cells infiltrating the injured lung not only degrade ATP to AMP and adenosine but also show enhanced NAD catabolism to ADPR involving CD38. Since CD38 competes with CD296 (ARTs) for the NAD substrate [45] the observed induction of *Cd38* may oppose NAD-induced apoptosis reported to be mediated by ARTs [46]. NAD is known to be released into the extracellular environment by injured or dead cells and to serve as danger signal that initiates immune responses [9]. NAD itself functions as substrate for ADP-ribosylation of cell surface molecules (such as CD45, CD8 and CD38) by ARTs [45] which has been shown to induce apoptosis in T cells [46], as stated above. ADPR, on the other hand, is a second messenger involved in T cell calcium signaling [47] and thereby T cell activation [48]. While we did not observe a further degradation of ADPR to AMP and adenosine, a recent study reported NAD as putative endogenous source of adenosine in human Jurkat T cells, a leukemia cell line [22]. Note, that all three murine T cell subsets in our study expressed the ectoenzyme *Pc-1* that is involved in the degradation of ADPR and AMP [9], [22], however, this did not translate into the formation of significant protein to permit the degradation of measurable amounts of ADPR to AMP. Obviously, human Jurkat T cells may differ from murine T cells in many respects [49], a phenomenon well known in the literature [50].

In summary, the present study provides first evidence that all T cell subsets infiltrating the LPS-injured lung show an enhanced ATP and NAD catabolism. While ATP is rapidly degraded to anti-inflammatory adenosine, NAD becomes converted to ADPR which is likely to counteract the ATP- and NAD- induced apoptosis and pro-inflammatory activation of T cells. The degradation products, adenosine and ADPR, can each affect cell function in an autocrine and/or paracrine manner. The balance between purinergic of P1 and P2 receptors during ALI is clearly shifted to the adenosine-A2a receptor which favors resolution of inflammation. The precise role of ADPR in the inflammatory processes of LPS-induced lung injury remains to be investigated.

## Supporting Information

Figure S1 Total number of immune cell subsets in the intravascular space (IV) under control conditions and 3 d and 7 d after induction of ALI and representative flow cytometry histograms of CD39 and CD73 expression. (A) Under unstressed conditions, lymphocytes represent the dominant cell type in the blood followed by monocytes and macrophages, granulocytes and NK cells. B cells, NK cells and T cells transiently decreased 3 d and re-increased 7 d after LPS exposure while granulocytes remained elevated. (B+C) Representative histograms of primary flow cytometric analysis for CD39 and CD73 expression as well as FMO controls. Data are mean ± SD (n = 5 mice per group). Statistical significance was assessed by one-way ANOVA with Dunnett's post hoc test. *P<0.05, **P<0.01, ***P<0.0001. ALI  =  acute lung injury, AM  =  alveolar macrophages, APC  =  antigen-presenting cells, BC  =  B cells, CTC  =  cytotoxic T cells, Gr  =  granulocytes, IV  =  intravascular space, LPS  =  lipopolysaccharide, n.d.  =  not detected, NKC  =  natural killer cells, SD  =  standard deviation, THC  =  T helper cells, Treg  =  regulatory T cells.
(TIF)

Figure S2 Percentage of CD39 and CD73 positive cells within and expression pattern of both ectoenzymes on various immune cell subsets from the IV under control conditions and 7 d after induction of ALI. (A+B) No significant change in the percentage of CD39 and CD73 expressing cells was found within the leukocyte subpopulations. (C+D) Expression levels of CD39 and CD73 assessed by means of the MFI were not different on the different immune cell subsets from the IV. Data are mean ± SD (n = 5 mice per group). Statistical significance was assessed by one-way ANOVA with Dunnett's post hoc test. *P<0.05, **P<0.01, ***P<0.0001. ALI  =  acute lung injury, AM  =  alveolar macrophages, APC  =  antigen-presenting cells, BC  =  B cells, CTC  =  cytotoxic T cells, Gr  =  granulocytes, IV  =  intravascular space, MFI  =  mean fluorescence intensity, M&M  =  monocytes and macrophages, n.d.  =  not detected, NKC  =  natural killer cells, SD  =  standard deviation, THC  =  T helper cells, Treg  =  regulatory T cells.
(TIF)

Figure S3 Gene expression of *Ada*, *Adk*, *Alp*, *Cd203*, and *Cx43* in T cell subsets isolated from the lung under basal conditions and 7 d after LPS exposure determined by quantitative *real-time* PCR. (A) Under basal condition *Alpl* and *Cd203* expression was not and *Cx43* barely detectable while *Ada* and *Adk* were moderately or low expressed in the T cell subsets. (B) Gene expression was not modulated by LPS exposure. Gene expression was normalized to beta-actin and relative expression levels are depicted. Data are mean ± SD (n = 4 mice per group). Statistical significance was assessed by Mann-Whitney U test.*P<0.05, **P<0.01, ***P<0.0001. Ada  =  adenosine deaminase, Adk  =  adenosine kinase, ALI  =  acute lung injury, Alp  =  alkaline phosphatase, Cx43  =  connexine 43, LPS  =  lipopolysaccharide, n.d.  =  not detected, SD  =  standard deviation.
(TIF)

Table S1 Overview on target genes that were measured using preloaded TaqMan Array Microfluidic Cards.
(DOCX)
