# Correction: Purinergic signaling on leukocytes infiltrating the LPS-injured lung

**DOI:** 10.1371/journal.pone.0106356

**Published:** 2014-08-19

**Authors:** The PLOS ONE Staff

There are errors in Figure 5(A) of the published paper. On the x-axis of the graph, sample name “Cd203” should read “Pc-1” and sample name “Art2.b” should read “Cd296”. Please see the corrected Figure 5 below.

**Figure 5:**
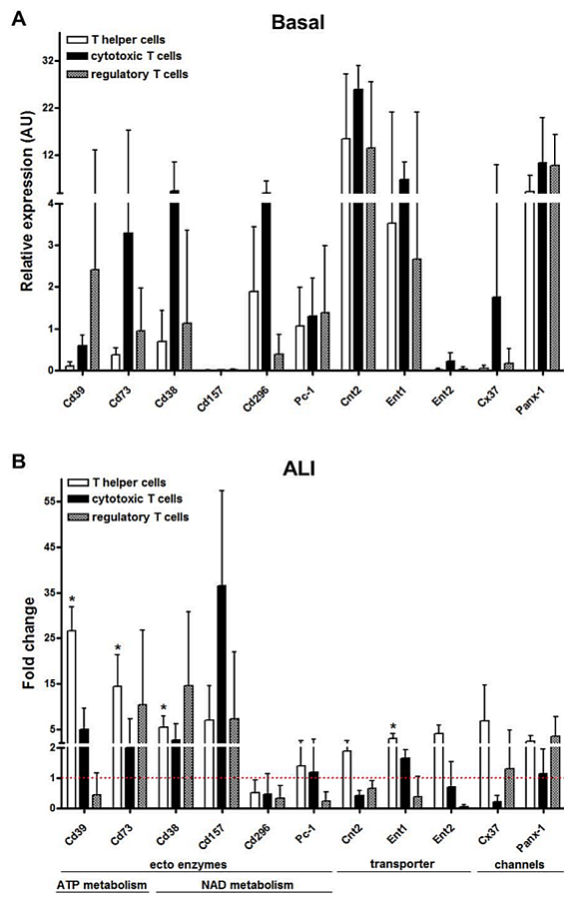
Gene expression of distinct ectoenzymes, transporters and channels in the T cell subsets isolated from lung tissue under basal conditions and 7*real-time* PCR. (A) Under basal conditions, T cell subsets expressed various ectoenzymes of ATP and NAD degradation cascade as well as nucleotide and nucleoside transporters and channels. Gene expression was normalized to beta-actin and relative expression levels are depicted. (B) In the diseased state, *Cd38*, *Cd39* and *Cd73*were significantly upregulated on T helper cells and tended to be increased in cytotoxic and regulatory T cells. *Ent1* transcripts were significantly increased in T helper cells. Data are mean ± SD (n  =  4 mice per group). Fold changes were calculated by comparing the expression under basal conditions to that in the injured lung 7 d post induction of ALI and statistical significance was then assessed by Mann-Whitney U test. *P<0.05, **P<0.01, ***P<0.0001. ALI  =  acute lung injury, Art2.b  =  ADP-ribosyltransferase 2b, ATP  =  adenosine triphosphate, AU  =  arbitrary units, Cnt  =  concentrative nucleoside transporter, Cx  =  connexin, Ent  =  equilibrative nucleoside transporter, LPS  =  lipopolysacch, NAD  =  nicotinamide adenine dinucleotide, Panx-1  =  pannexin-1, SD  =  standard deviation.
