# Supplementary figures and images for: Purinergic Signaling on Leukocytes Infiltrating the LPS-Injured Lung
Source: PLoS One. 2014 Apr 18;9(4):e95382. doi: 10.1371/journal.pone.0095382 (PMC3991673; doi:10.1371/journal.pone.0095382)

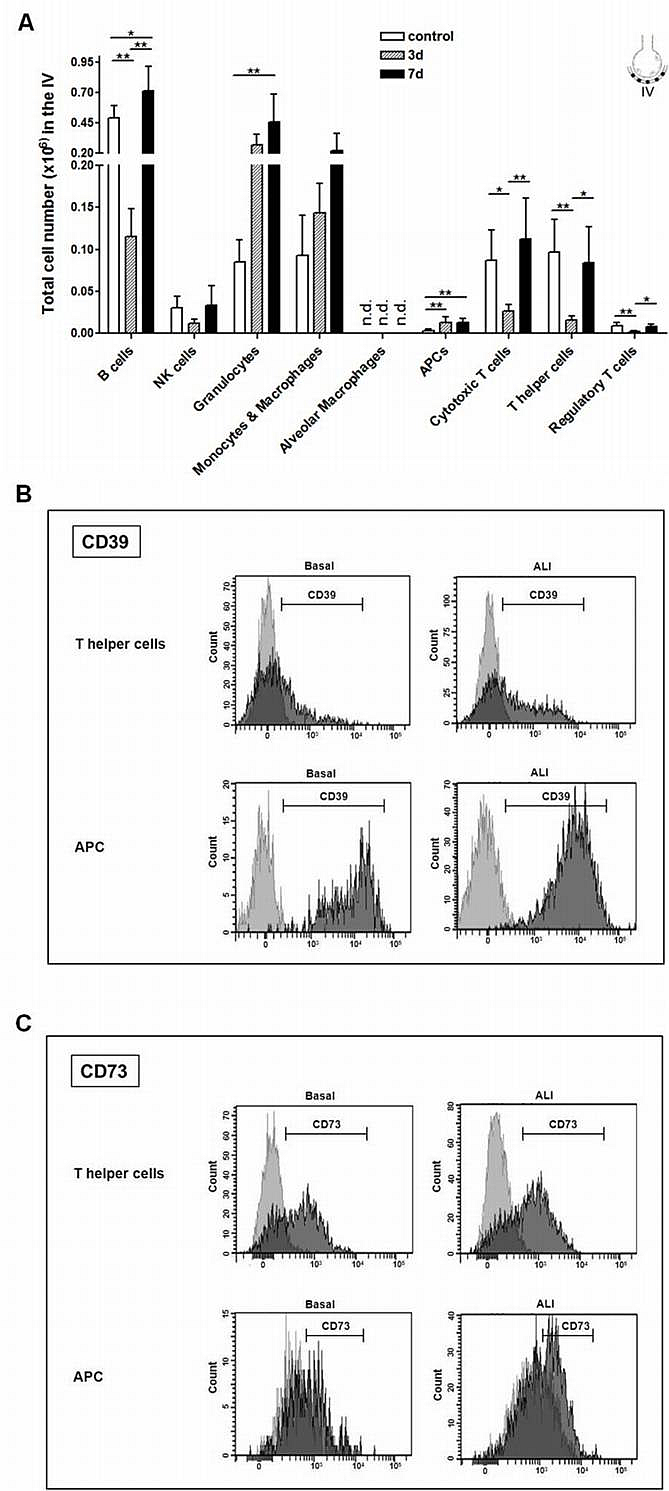

Supplement: Figure S1 — Total number of immune cell subsets in the intravascular space (IV) under control conditions and 3 d and 7 d after induction of ALI and representative flow cytometry histograms of CD39 and CD73 expression. (A) Under unstressed conditions, lymphocytes represent the dominant cell type in the blood followed by monocytes and macrophages, granulocytes and NK cells. B cells, NK cells and T cells transiently decreased 3 d and re-increased 7 d after LPS exposure while granulocytes remained elevated. (B+C) Representative histograms of primary flow cytometric analysis for CD39 and CD73 expression as well as FMO controls. Data are mean ± SD (n = 5 mice per group). Statistical significance was assessed by one-way ANOVA with Dunnett's post hoc test. *P<0.05, **P<0.01, ***P<0.0001. ALI = acute lung injury, AM = alveolar macrophages, APC = antigen-presenting cells, BC = B cells, CTC = cytotoxic T cells, Gr = granulocytes, IV = intravascular space, LPS = lipopolysaccharide, n.d. = not detected, NKC = natural killer cells, SD = standard deviation, THC = T helper cells, Treg = regulatory T cells. (TIF) [file pone.0095382.s001.tif]

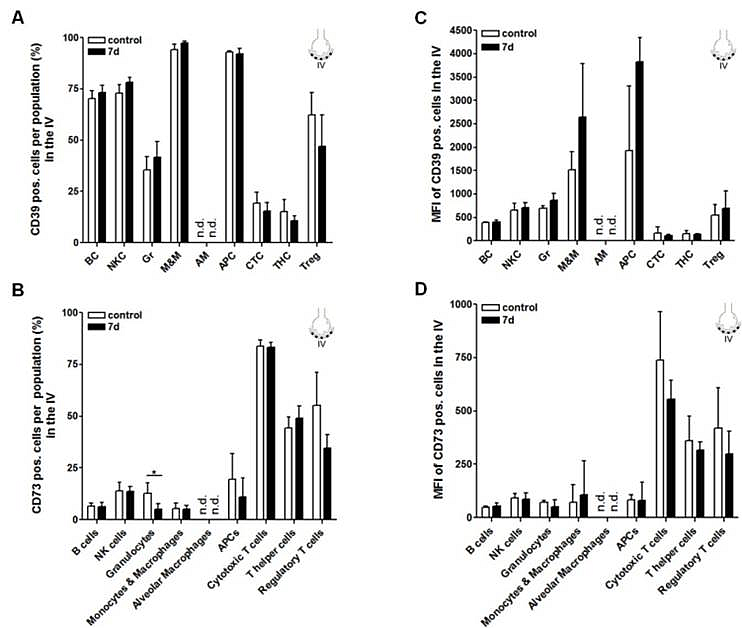

Supplement: Figure S2 — Percentage of CD39 and CD73 positive cells within and expression pattern of both ectoenzymes on various immune cell subsets from the IV under control conditions and 7 d after induction of ALI. (A+B) No significant change in the percentage of CD39 and CD73 expressing cells was found within the leukocyte subpopulations. (C+D) Expression levels of CD39 and CD73 assessed by means of the MFI were not different on the different immune cell subsets from the IV. Data are mean ± SD (n = 5 mice per group). Statistical significance was assessed by one-way ANOVA with Dunnett's post hoc test. *P<0.05, **P<0.01, ***P<0.0001. ALI = acute lung injury, AM = alveolar macrophages, APC = antigen-presenting cells, BC = B cells, CTC = cytotoxic T cells, Gr = granulocytes, IV = intravascular space, MFI = mean fluorescence intensity, M&M = monocytes and macrophages, n.d. = not detected, NKC = natural killer cells, SD = standard deviation, THC = T helper cells, Treg = regulatory T cells. (TIF) [file pone.0095382.s002.tif]

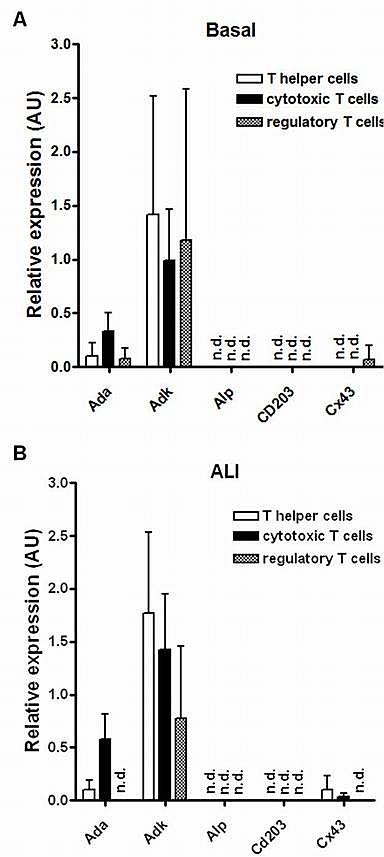

Supplement: Figure S3 — Gene expression of Ada, Adk, Alp, Cd203, and Cx43 in T cell subsets isolated from the lung under basal conditions and 7 d after LPS exposure determined by quantitative real-time PCR. (A) Under basal condition Alpl and Cd203 expression was not and Cx43 barely detectable while Ada and Adk were moderately or low expressed in the T cell subsets. (B) Gene expression was not modulated by LPS exposure. Gene expression was normalized to beta-actin and relative expression levels are depicted. Data are mean ± SD (n = 4 mice per group). Statistical significance was assessed by Mann-Whitney U test.*P<0.05, **P<0.01, ***P<0.0001. Ada = adenosine deaminase, Adk = adenosine kinase, ALI = acute lung injury, Alp = alkaline phosphatase, Cx43 = connexine 43, LPS = lipopolysaccharide, n.d. = not detected, SD = standard deviation. (TIF) [file pone.0095382.s003.tif]
