# Supplementary material for: Purinergic Signaling on Leukocytes Infiltrating the LPS-Injured Lung
Source: PLoS One. 2014 Apr 18;9(4):e95382. doi: 10.1371/journal.pone.0095382 (PMC3991673; doi:10.1371/journal.pone.0095382)
Supplement: Table S1 — Overview on target genes that were measured using preloaded TaqMan Array Microfluidic Cards. (DOCX) [file pone.0095382.s004.docx]

**Table S1**

**Table S1:** Overview on target genes that were measured using preloaded TaqMan Array Microfluidic Cards

| **Function** | **Gene symbol** | **Gene name** |
| --- | --- | --- |
| housekeeper | Actn | beta-actin |
|  | Tbp | TATA-box binding protein |
| internal control | 18S rRNA | 18S ribosomal RNA |
| enzymes | Cd38 | ADP-ribosyl cyclase/cyclic ADP-ribose hydrolase |
|  | Cd39/Entpd1 | ectonucleoside triphosphate diphosphohydrolase |
|  | Cd73/5'-Nte | ecto-5'-nucleotidase |
|  | Cd157/Bst-1 | bone marrow stromal cell antigen 1 |
|  | Cd296 /Art2b | ADP-ribosyltransferase 2 |
|  | Pc-1/Enpp1 | ectonucleotide pyrophosphatase/phosphodiesterase 1 |
|  | CD203c/Enpp3 | ectonucleotide pyrophosphatase/phosphodiesterase 3 |
|  | Ada | adenosine deaminase |
|  | Adk | adenosine kinase |
|  | Alp | alkaline phosphatase |
| adenosine receptors | A1/Adora1 | adenosine A1 receptor |
|  | A2a/Adora2a | adenosine A2a receptor |
|  | A2b/Adora2b | adenosine A2b receptor |
|  | A3/Adora3 | adenosine A3 receptor |
| P2x receptors | P2x1 | purinergic receptor P2X, ligand-gated ion channel, 1 |
|  | P2x4 | purinergic receptor P2X, ligand-gated ion channel, 4 |
|  | P2x5 | purinergic receptor P2X, ligand-gated ion channel, 5 |
|  | P2x7 | purinergic receptor P2X, ligand-gated ion channel, 7 |
| P2y receptors | P2y1 | purinergic receptor P2Y, G-protein coupled, 1 |
|  | P2y2 | purinergic receptor P2Y, G-protein coupled, 2 |
|  | P2y4 | purinergic receptor P2Y, G-protein coupled, 4 |
|  | P2y6 | purinergic receptor P2Y, G-protein coupled, 6 |
| channels | Cx37 | connexin 37 |
|  | Cx43 | connexin 43 |
|  | Panx-1 | pannexin 1 |
| transporters | Ent1/SLC29a1 | equilibrative nucleoside transporter 1 |
|  | Ent2/SLC29a2 | equilibrative nucleoside transporter 2 |
|  | Cnt2/SLC28a2 | concentrative nucleoside transporter 2 |
